# Dissecting the Impact of Genetic Background on Oncogenic Response to Radiation Exposure in the *Ptch1*^+/−^ Mouse Model

**DOI:** 10.3390/cells13221912

**Published:** 2024-11-19

**Authors:** Barbara Tanno, Emiliano Fratini, Simona Leonardi, Flavia Novelli, Valentina Pisano, Mariateresa Mancuso, Simonetta Pazzaglia

**Affiliations:** Division of Biotechnologies, Italian National Agency for New Technologies, Energy and Sustainable Economic Development (ENEA), Via Anguillarese 301, 00123 Rome, Italy; emiliano.fratini@enea.it (E.F.); simona.leonardi@enea.it (S.L.); flavia.novelli@enea.it (F.N.); vpisano93@yahoo.it (V.P.); mariateresa.mancuso@enea.it (M.M.)

**Keywords:** medulloblastoma mouse model, DNA damage, DNA repair, apoptosis, cell cycle, gene expression, stemness, irradiation

## Abstract

Medulloblastoma (MB) is a common primary brain cancer in children. The sonic hedgehog (SHH) pathway is indispensable for the normal development of the cerebellum, and MB is often caused by persistent SHH activation owing to mutations in pathway components. Patched1 (*PTCH1*) is the primary receptor for the SHH ligand and a negative regulator of the SHH signal transduction pathway. Mice heterozygous for the *Ptch1* gene (*Ptch1*^+/−^) are predisposed to MB development. Irradiation of newborn *Ptch1*^+/−^ mice dramatically increases MB occurrence. A genetic background carrying the *Ptch1* mutation significantly influences the risk of developing MB. This study aims to investigate the genetic background-related mechanisms that regulate radiation-induced cellular response and oncogenesis in the cerebellum. We employed multiple approaches, including: (a) analysis of cellular radiosensitivity in granule cell precursors (GCPs), the MB cells of origin, derived from *Ptch1* mice with a genetic background that is sensitive (CD1) or resistant (C57Bl/6) to the induction of radiogenic MB; (b) identification of genes differentially expressed in spontaneous and radiation-induced MBs from these two mouse strains; (c) bioinformatic analysis to correlate the expression of radiation-induced genes with survival in MB patients; and (d) examining the expression of these genes in ex vivo MBs induced by single or repeated radiation doses. We have identified a potential gene expression signature—*Trp53bp1*, *Bax*, *Cyclin D1*, *p21*, and *Nanog*—that influences tumor response. In ex vivo cultured spontaneous MBs, the expression levels of these genes increase after irradiation in CD1 mice, but not in mice with a C57Bl/6 genetic background, suggesting that this signature could predict tumor response to radiation therapy and help develop strategies for targeting DNA damage repair in tumors. A detailed understanding of the mechanisms behind genetic background-related susceptibility to radiation-induced oncogenic responses is crucial for translational research.

## 1. Introduction

Medulloblastomas (MBs) are the most common primary central nervous system tumors in childhood, thought to originate from a specific group of cells called precursors of the granule cells (GCPs) in the developing cerebellum [1,2]. Between the second and fourth postnatal days (P2–P4) in mice, various signaling pathways stimulate the proliferation of GCPs. Among these, the Sonic hedgehog (Shh) pathway plays a key role in significantly increasing the GCP population during the early postnatal period [3,4]. Importantly, abnormalities in the Shh pathway predispose both mice and humans to MB. Mice with genetic mutations constitutively activating the Shh pathway are prone to developing MB [5,6,7], and SHH-activated MBs make up a significant proportion of MB cases in patients [1].

The Shh pathway is crucial for the development of almost all organs and for maintaining postnatal homeostasis and regeneration. This pathway transmits signals through two transmembrane proteins, Smoothened (SMO) and its negative regulator, Patched1 (PTCH1). Heterozygous germline mutations in the *PTCH1* gene in humans cause Gorlin syndrome, also known as nevoid basal cell carcinoma syndrome. This rare autosomal dominant disorder is characterized by multiple clinical manifestations, including the early onset of cutaneous basal cell carcinomas (BCCs). Remarkably, mice with a single functional copy of the *Ptch1* gene (*Ptch1*^+/−^) recapitulate most features of Gorlin syndrome, including susceptibility to MB and other tumors development, as well as radiation hypersensitivity [5,6,8,9]. Neonatal irradiation dramatically increases the occurrence of BCCs and MBs in *Ptch1*^+/−^ mice [9,10].

The genetic background hosting the *Ptch1* mutation can dramatically alter the individual risk for developing tumors, and this incomplete mutation penetrance suggests that other signaling molecules cooperate with Shh to enhance tumor formation. In the *Ptch1*^+/−^ mouse model, higher spontaneous MB incidence is shown in a C57BL/6 background compared with a CD1 background (40.9% vs. 7.7%) [11,12], suggesting that a larger proportion of dividing cells may acquire tumor-initiating mutations in the early postnatal cerebellum depending on the genetic background [11]. However, while irradiation of newborn *Ptch1*^+/−^ mice with a CD1 background with a single dose of 3 Gy X-rays dramatically increased MBs over the spontaneous rate [9], no increase in MB incidence was observed in *Ptch1*^+/−^ mice bred with a C57BL/6 background after neonatal irradiation [11]. These findings indicate that a CD1 background can be considered permissive, or that C57BL/6 can be considered suppressive, to radiogenic MBs, and that different sets of genes may control susceptibility to spontaneous and radiation-induced MBs.

Understanding how individual susceptibility to radiation affects health outcomes remains a top priority in radiation protection [13]. This study employs multiple approaches to investigate the genetic background-related mechanisms that regulate radiation-induced cellular response and oncogenesis. These approaches include: (i) the analysis of endpoints and characteristic mechanisms of cellular radiosensitivity in GCPs isolated from *Ptch1*^+/−^ mice bred with CD1 or C57Bl/6 backgrounds; (ii) the search for differentially expressed genes in spontaneous and radiation-induced MBs from the two mouse lines, including genes associated with DNA damage (*γ-H2AX* and *Trp53bp1*) and DNA damage response (DDR) pathways (*p53*, *p21*, *p16*, and *Bax*), as well as cell cycle regulation and stemness markers; (iii) the use of bioinformatics analysis to correlate the expression levels of identified radiation-induced genes with survival outcomes in MB patients; and (iv) the analysis of the identified genes in ex vivo MBs after single or repeated irradiation fractions.

A mechanistic understanding of susceptibility to radiation oncogenic responses might have important implications in translational research and is crucial for developing personalized and effective treatment strategies for cancer patients.

## 2. Materials and Methods

### 2.1. Mice and Cell Cultures 

Mice lacking one *Ptch1* allele were maintained on CD1 (CD1*^Ptch1^*^+/−^ [CD1.129-Ptch1^tm1Zim^/Cnrm]) or C57Bl/6 (C57Bl/6*^Ptch1^*^+/−^ [B6.129-Ptch1^tm1Zim^/Cnrm]) backgrounds [6,11]. Throughout the experimental duration, animals were housed under conventional conditions with food and water available ad libitum and a 12 h light–dark cycle.

For in vitro investigations, GCPs were purified from CD1*^Ptch1^*^+/−^ and C57Bl/6*^Ptch1^*^+/−^ mouse cerebella at P2 and maintained in culture as described [14]. They were cultured in a Neurobasal medium with penicillin–streptomycin and L-Glutamine plus a B27 supplement without vitamin A. Unless otherwise indicated, media and supplements for cell culture were purchased from Gibco-Invitrogen (Carlsbad, CA, USA) and chemicals were purchased from Sigma-Aldrich (St. Louis, MO, USA). Cultured GCPs were exposed to doses of 2 Gy or 6 Gy of X-rays, using a Gilardoni CHF 320 G X-ray generator (Gilardoni S.p.A., Mandello del Lario, Italy) operated at 250 kVp, with HVL = 1.6 mm Cu (additional filtration of 2.0 mm Al and 0.5 mm Cu), or left untreated. For growth kinetics, GCPs from CD1*^Ptch1^*^+/−^ and C57Bl/6*^Ptch1^*^+/−^ mice were seeded at clonal density (28,000 cells/cm^2^). On the following day, they were irradiated or left untreated. Cells were counted at fixed days (1, 5, 8, and 9).

For ex vivo investigations on the radiation response of MBs to a single or two repeated X-ray treatments, spontaneous MBs (n = 4 for each group) from C57Bl/6*^Ptch1^*^+/−^ and CD1*^Ptch1^*^+/−^ mice were explanted and cultured in Ultraculture medium (Lonza Walkersville Inc., Walkersville, MD, USA), supplemented with 20 ng/mL EGF, 20 ng/mL bFGF (Peprotech, Cranbury, NJ, USA), penicillin–streptomycin, L-Glutamine, and a B27 supplement without vitamin A, and 5 mM Sag (Smoothened agonist; Sigma-Aldrich, St. Louis, MO, USA). MB cells were seeded and exposed to 2 Gy of X-rays. They were then either collected 3 days later or given a second 2 Gy dose and collected 3 days after the final exposure. 

For the in vivo and ex vivo analysis of gene expression, radiogenic and spontaneous MBs were explanted from irradiated or unexposed CD1^*Ptch1*+/−^ and C57Bl/6*^Ptch1^*^+/−^ mice and snap frozen for subsequent q-PCR analysis.

### 2.2. Silencing of Nanog and Oct-4 in GCPs and Neurospheres Assay

GCPs from CD1*^Ptch1^*^+/−^ and C57Bl/6*^Ptch1^*^+/−^ mice were transfected with siRNA duplexes (40 nM) directed against *Nanog* and *Oct-4* mRNA coding sequences [siGENOME Mouse Nanog siRNA SMART Pool, siGENOME Mouse Oct-4 Dharmacon Inc. Lafayette, CO, USA] using the INTERFERin™ siRNA Transfection Reagent (Polyplus, Illkirich, France) according to the manufacturer’s instructions. Control transfections were carried out with a pool of validated siRNA controls (siGENOME Non-Targeting siRNA Pool #1, Dharmacon). One day after transfection, GCPs were seeded for the neurosphere assay and irradiated with 2 Gy.

For the neurosphere-forming assay, cells were plated at clonal density (1–2 cells/mm^2^) into 96-well plates and cultured in a selective medium, DMEM/F12 supplemented with 0.6% glucose, 25 μg/mL insulin, 60 μg/mL N-acetyl-L-cysteine, 2 μg/mL, heparin, 20 ng/mL EGF, 20 ng/mL bFGF, penicillin–streptomycin, and a B27 supplement without vitamin A. For the morphometric analysis of neurospheres, images were captured with a Leica digital camera and analyzed using the image analysis software LASCore (Leica Application Suite Version 4.3, Leica Microsystems, Milan, Italy).

### 2.3. Flow Cytometry Analysis

For cell cycle analysis, propidium iodide staining was performed using 1 × 10^6^ fixed cells (Fixation/Permeabilization solution BD Biosciences, San Jose, CA, USA), incubated at 4 °C. Cells were centrifuged, and pellets were suspended in PI/RNase staining buffer (BD Biosciences, Franklin Lakes, NJ, USA). Cells were incubated for 5 min at room temperature using a coulter flow cytometer (CytoFLEX S, Beckman, Indianapolis, IN, USA).

For γ-H2AX staining, 2 × 10^6^ cells were fixed (Fixation/Permeabilization solution, BD Biosciences, Franklin Lakes, NJ, USA) and incubated at 4 °C. For the endogenous visualization of γ-H2AX by CytoFLEX, we used anti-phospho γ-H2AX (Ser139) (Cell Signaling Technology, Inc., Danvers, MA, USA). The primary antibody was prepared in 0.5% BSA (Sigma-Aldrich, St. Louis, MO, USA) PBS buffer, incubated for 1 h on ice, and washed in 1X PBS. The fluorochrome-conjugated secondary antibody was diluted (in 3% BSA/PBS) and incubated for 1 h on ice, washed, and analyzed by flow cytometry using CytoFLEX. 

For Annexin V staining, we used an Annexin V-FITC Early Apoptosis Detection Kit (Cell Signaling Technology, Danvers, MA, USA), following the manufacturer’s instructions. Cell cycle distribution and hypo diploid DNA content, as well as γ-H2AX and Annexin V staining, were evaluated by FCS express Cytometry 7 plus (version 7.12.0007) (DeNovo Software, Pasadena, CA, USA).

### 2.4. p53 Functional Test 

GCPs from CD1*^Ptch1^*^+/−^ and C57Bl/6*^Ptch1^*^+/−^ mice were seeded and transfected with a Nanoluc-*p*53 Response Element (p53 RE) pNL vector (Promega Corporation, Madison, WI, USA) using Lipofectamine 2000 (Thermofisher scientific, Waltham, MA, USA). The following day, GCPs were irradiated with 2 Gy of X-rays or left untreated. At 2 h post-irradiation, we measured firefly and NanoLuc luciferase activities in single samples. We used the Nano-Glo^®^ Dual-Luciferase^®^ Reporter Assay System (Promega Italia Srl, Milan, Italy), following the manufacturer’s instructions.

### 2.5. RNA Isolation and Real-Time qPCR 

RNA isolation from GCPs and MBs (obtained from the ENEA archive of frozen tumors) was performed with a miRNeasy Mini Kit (QIAGEN, Hilden, Germany). A total of 2 μg of total RNA was reverse transcribed with a High-Capacity cDNA Reverse Transcription Kit (Applied Biosystems, Foster City, CA, USA), and qPCR reactions were performed in triplicate from each biological replicate in the QuantStudio™ 5 Real-Time PCR System (Applied Biosystems) using the Power up SYBR^®^ Green PCR Master Mix (Applied Biosystems). Relative quantification was carried out using the ΔΔCt method, with Glyceraldehyde-3-phosphate (*Gapdh*) as the endogenous housekeeping control. The oligonucleotide primers used for quantitative RT-PCR are listed below.**Gene****Forward Primer****Reverse Primer***Oct-4*5′-AAAGCCCTGCAGAAGGAGCTAGAA-3′5′-AACACCTTTCCAAAGAGAACGCCC-3′*Nanog*5′-AAGCAGAAGATGCGGACTG-3′5′-GCTTGCACTTCATCCTTTGG-3′*p53*5′-TGCATGGACGATCTGTTGCT-3′5′-TTCACTTGGGCCTTCAAAAAA-3′*p21*5′-CGAGAACGGTGGAACTTTGAC-3′5′-CAGGGCTCAGGTAGACCTTG-3′*Trp53bp1*5′-TCACTGCCATGGAGGAGC-3′5′-GGATGCCTGGTACTGTTTGG-3′*Cyclin-D1*5′-TCCGCAAGCATGCACAGA-3′5′-AGGGTGGGTTGGAAATGAACT-3′*Bax*5′-ATCCAGGATCGAGCAGGGCG-3′5′-ACTCGCTCAGCTTCTTGGTG-3′*p16*5′-CCGAACTCTTTCGGTCGTAC-3′5′-AGTTCGAATCTGCACCGTAGT-3′*Gapdh*5′-CATGGCCTTCCGTGTTCCTA-3′5′-GCGGCACGTCAGATCCA-3′

### 2.6. Bioinformatics Analysis 

Dataset interrogation was conducted on the R2 Genomic Analysis and Visualization Platform (Amsterdam UMC, https://hgserver1.amc.nl/cgi-bin/r2/; accessed on 16 July 2024) to perform a Kaplan–Meier survival analysis. An observational experiment on the RNA-seq profile of 331 primary tumors from patients diagnosed with MB was selected [15]. Survival analysis was performed using the R2 KaplanScanner tool to find the optimal 2-group segregation based on gene expression (Figure S1). Kaplan–Meier plots were generated from two groups (high and low gene expression) for the following genes: *TP53BP1*, *BAX*, *CYCLIN D1*, *P21*, *P16*, *OCT-4*, and *NANOG*.

### 2.7. Statistical Analysis 

Statistical tests were performed with Graph Pad Prism v.7 software. The *p*-values were determined using a non-parametric two-tailed *t*-test or an Anova test. * *p* < 0.05, ** *p* < 0.01, *** *p* < 0.001, and **** *p* < 0.0001. Results are expressed as average biological replicates ± SD.

For survival distributions, *p*-values were determined using a log-rank test on the R2 Genomic Analysis and Visualization Platform.

## 3. Results

### 3.1. DNA Damage Response in GCPs from Ptch1^+/−^ Mice

GCPs play a pivotal role in cerebellar development and are considered the potential cells of origin for some MB subtypes, including the Shh-dependent subtype. To investigate the mechanisms of GCP radioresistance/radiosensitivity, we performed a comparative gene expression analysis on GCPs from *Ptch1*^+/−^ mice bred on different genetic backgrounds (CD1 and C57Bl/6). GCPs were purified from the mouse cerebellum on postnatal day 2 (P2).

**Figure 1:**
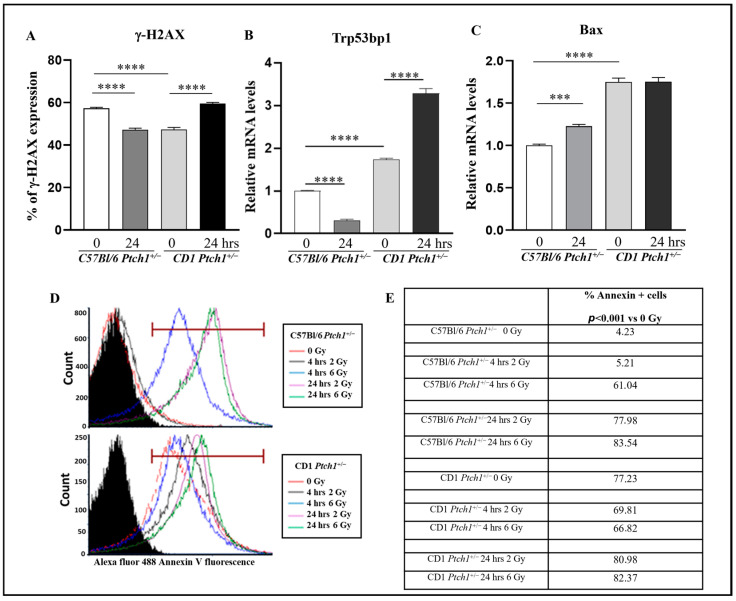
Characterization of residual DSBs and apoptosis in irradiated GCPs^C57Bl-^*^Ptch1^*^+/−^ and GCPs^CD1-^*^Ptch1^*^+/−^ at 24 h post-irradiation and the relatively unexposed controls. (**A**) Flow cytometry analysis of γH2AX in GCPs. (**B**,**C**) mRNA expression level of *Trp53bp1* and *Bax*. Evaluation of apoptosis by Annexin V assay: (**D**) plots and (**E**) table. Results are expressed as an average of 3 biological replicates ± SD; in qPCR, untreated GCPs^C57Bl-^*^Ptch1^*^+/−^ were taken as 1. Differences between irradiated and unirradiated GCPs of each genotype were analyzed with a Student’s *t*-test: show **** *p* < 0.0001, *** *p* < 0.001.

We quantified the expression of γ-H2AX, a double-strand break (DSB) marker, by flow cytometric analysis of control or irradiated GCPs (24 h post 2 Gy of X-rays). High γ-H2AX levels were detected in unirradiated GCPs^CD1-*Ptch1*+/−^ and GCPs^C57Bl-^*^Ptch1^*^+/−^, with a significantly higher frequency in GCPs^C57Bl-^*^Ptch1^*^+/−^ (57.8% vs. 47.96%; *p* < 0.001). While irradiated GCPs^CD1-*Ptch1*+/−^ showed significantly increased percentages of γ-H2AX-positive cells compared to their control counterparts (59.37% vs. 47.96%; *p* < 0.001; Figure 1A), a reverse pattern was observed in irradiated GCPs^C57Bl-^*^Ptch1^*^+/−^, which showed significantly lower percentages of γ-H2AX-positive cells compared to matching control GCPs (47.19% irradiated vs. 57.8% unirradiated cells, *p* < 0.001).

Next, we analyzed the expression of Trp53bp1, another DSB marker involved in the recruitment of DNA repair factors. Mice with unirradiated GCPs^CD1-*Ptch1*+/−^ exhibited significantly higher expression of Trp53bp1 compared to GCPs^C57Bl-^*^Ptch1^*^+/−^ (1.7-fold upregulation, *p* < 0.0001). However, mirroring γ-H2AX, the expression of Trp53bp1 in irradiated GCPs^C57Bl-^*^Ptch1^*^+/−^ was significantly lower at 24 h post-irradiation compared to unirradiated GCPs (0.31 vs. 1, *p* < 0.0001; Figure 1B). The decreased residual DSB rate in irradiated GCPs^C57Bl-^*^Ptch1^*^+/−^ was detected with two different DSB markers and techniques, underscoring the reliability and robustness of these results.

To further explore GCP radiation responses, we conducted a gene expression analysis of Bax, a pro-apoptotic factor (Figure 1C). Unirradiated GCPs^CD1-*Ptch1*+/−^ exhibited a 70% higher level of Bax mRNA compared to unirradiated GCPs^C57Bl-^*^Ptch1^*^+/−^ (*p* < 0.0001; Figure 1C). Furthermore, irradiation induced a significant 20% increase in Bax mRNA in GCPs^C57Bl-^*^Ptch1^*^+/−^ (*p* = 0.0001), while no changes were observed in irradiated GCPs^CD1-*Ptch1*+/−^ compared to matching controls. To determine whether Bax deregulation caused cell death, we evaluated apoptosis by Annexin V in GCPs^C57Bl-^*^Ptch1^*^+/−^ and GCPs^CD1-*Ptch1*+/−^ at 4 h and 24 h post-irradiation and in unirradiated GPCs of both genotypes. We show a dramatic basal difference of 17-fold in the number of Annexin V+ cells between unexposed GCPs^C57Bl-^^*Ptch1*+/−^ and GCPs^CD1-*Ptch1*+/−^ (4.23% vs. 72.23%; Figure 1D,E). Consistent with the radiation-induced increase in Bax expression, the apoptotic rate in GCPs^C57Bl-^*^Ptch1^*^+/−^ progressively increased following irradiation, reaching an 18-fold increase at 24 h post-irradiation compared to their unirradiated counterparts (77.78% vs. 4.23%, *p* < 0.001). Furthermore, in GCPs^C57Bl-^*^Ptch1^*^+/−^, we show that radiation dose influences the kinetics of cell death, as at 4 h post-irradiation with 6 Gy, the onset of apoptosis was accelerated, and a 14-fold increase in Annexin V+ cells was already observed (61.04% vs. 4.23%, *p* = 0.001). On the other hand, GCPs^CD1-*Ptch1*+/−^ only showed 1.12-fold and 1.14-fold increases at 2 Gy and 6 Gy, respectively, at 24 h post-irradiation (Figure 1D,E).

Overall, these findings, which indicate marked genetic background-related differences in DNA damage response and apoptosis in GCPs, suggest that differential processing of radiation-induced DNA damage may underline the opposite strain-related radiosensitivity to MB induction.

### 3.2. Effects of Irradiation on GCP Growth Kinetics and Expression of Stemness Genes 

**Figure 2:**
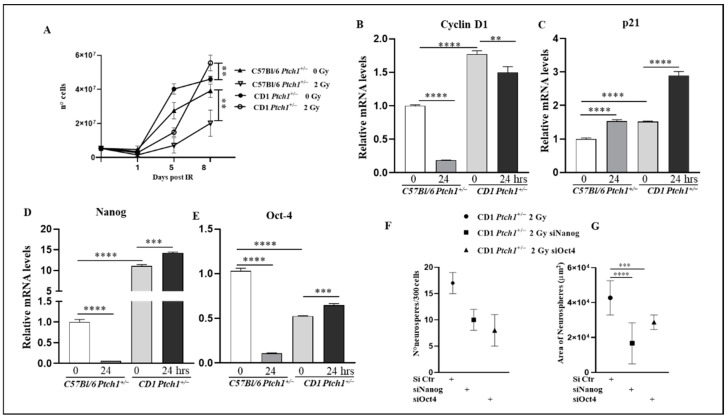
Characterization of growth kinetics, cell cycle, and stemness in GCPs from irradiated CD1*^Ptch1^*^+/−^ and C57Bl/6*^Ptch1^*^+/−^ mice and relative unexposed controls. (**A**) Growth curves of GCPs, in irradiated and unirradiated conditions for 8 days. (**B**) Evaluation of the expression of *Cyclin D1* mRNA at 24 h post-irradiation and (**C**) *p21* (**D**) *Nanog*, and (**E**) *Oct-4*. Neurosphere assay showing quantification (**F**) and size measurements (**G**) of neurospheres derived from irradiated GCPs after *Nanog* and *Oct-4* inhibition. The results are expressed as an average of 3 biological replicates ± SD; untreated GCPs^C57Bl-^*^Ptch1^*^+/−^ were taken as 1. The differences between the irradiated and corresponding unirradiated conditions of each genotype were analyzed with a Student’s *t*-test: show **** *p* < 0.0001, *** *p* < 0.001, and ** *p* < 0.01.

We then investigated the growth kinetics of GCPs^C57Bl-^*^Ptch1^*^+/−^ and GCPs^CD1-*Ptch1*+/−^ irradiated with 2 Gy or left untreated. Seeding of 5 × 10^3^ GCPs was performed, followed by irradiation, and the cell numbers were assessed at various post-irradiation time points (Figure 2A). At 5 days post-irradiation, both GCP populations exhibited a significant decrease in cell numbers compared to their respective unirradiated counterparts (−75% for GCPs^C57Bl-^*^Ptch1^*^+/−^, *p* = 0.008; −60% for GCPs^CD1-*Ptch1*+/−^, *p* = 0.0001). However, by 8 days post-irradiation, the number of irradiated GCPs^C57Bl-^*^Ptch1^*^+/−^ remained lower than that of unirradiated cells (−50%, *p* = 0.0047). In contrast, GCPs^CD1-*Ptch1*+/−^ exhibited increased recovery and proliferation rates compared to the unirradiated population (+20%, *p* = 0.0086).

To further characterize the radiation response, we evaluated the expression of cell cycle regulators. The baseline expression of *Cyclin D1* mRNA, a gene involved in cell cycle regulation, was found to be higher in GCPs^CD1-*Ptch1*+/−^ than in GCPs^C57Bl-^*^Ptch1^*^+/−^ (*p* < 0.001). However, consistent with the growth kinetics data indicating a significant radiation-induced growth delay in GCPs^C57Bl-^*^Ptch1^*^+/−^, there was a notable 80% reduction in the expression level at 24 h post-irradiation in GCPs^C57Bl-^*^Ptch1^*^+/−^ (*p* < 0.0001). In contrast, GCPs^CD1-*Ptch1*+/−^ exhibited a smaller decrease of 20% (*p* < 0.001) at the same time point (Figure 2B). We also evaluated expression levels of *p21*, a cell cycle inhibitor required for progression through the G1 phase. Basal *p21* expression levels in unirradiated GCPs^C57Bl-^*^Ptch1^*^+/−^ were significantly lower compared to GCPs^CD1-*Ptch1*+/−^ (*p* < 0.0001). Irradiation induced significant increases of 35% and 48% in irradiated GCPs^C57Bl-^*^Ptch1^*^+/−^ (*p* < 0.0001) and GCPs^CD1-*Ptch1*+/−^ (*p* < 0.0001) compared to matching controls (Figure 2C).

Our previous work demonstrated that the activation of stemness genes is responsible for radiation-induced Shh-mediated MB tumorigenesis in irradiated GCPs^CD1-*Ptch1*+/−^ [14]. Therefore, we compared the expression of the stem cell transcription factors *Nanog* and *Oct-4* in GCPs^C57Bl-^*^Ptch1^*^+/−^ and GCPs^CD1-*Ptch1*+/−^. The baseline expression of *Nanog* mRNA was 15 times higher in GCPs^CD1-*Ptch1*+/−^ compared to GCPs^C57Bl-^*^Ptch1^*^+/−^ (*p* < 0.0001), while *Oct-4* mRNA was 2-fold lower in GCPs^CD1-*Ptch1*+/−^ (*p* < 0.0001) (Figure 2D,E). In response to irradiation, GCPs^C57Bl-^*^Ptch1^*^+/−^ exhibited a substantial decrease in both *Nanog* and *Oct-4* expression levels compared to their unirradiated counterparts (~94% and 90%, respectively, *p* < 0.0001). Conversely, irradiated GCPs^CD1-*Ptch1*+/−^ showed a significant increase in both genes (*Nanog* 28% and *Oct-4* 23%, *p* = 0.0001) compared to matching controls (Figure 2D,E).

To further investigate the roles of stemness genes in the ionizing radiation response of GCPs, we conducted a neurosphere assay under irradiated conditions following the silencing of *Nanog* and *Oct-4* genes. GCPs were purified and cultured at clonal density, and both GCPs^C57Bl-^*^Ptch1^*^+/−^ and GCPs^CD1-*Ptch1*+/−^ were assessed for the number and size of neurospheres at 12 days post-irradiation. Mice with GCPs^CD1-*Ptch1*+/−^ formed an average of 17 final colonies, with a mean area of 4.2 × 10^4^ μm^2^, and the GCPs^C57Bl-^*^Ptch1^*^+/−^ did not develop neurospheres (data not shown). Additionally, when either *Nanog* or *Oct-4* genes were silenced, a tendency towards reduced neurosphere numbers (indicative of diminished cell reprogramming) was observed in GCPs^CD1-*Ptch1*+/−^ (siNanog 41%, *p* = 0.0728; siOct-4 53%, *p* = 0.0717) (see Figure 2F). Furthermore, *Nanog* or *Oct-4* gene silencing led to a significant decrease of 61% (*p* < 0.0001) and 33% (*p* = 0.0008) in the size of GCP neurospheres compared to their intact counterparts (Figure 2G), indicating their relevance in the maintenance of self-renewal.

Together, these results highlight significant genetic background-related opposite responses of GCPs to irradiation, impacting growth kinetics and stemness. This suggests that radiation-induced amplification of the stem-like cell population could contribute to the extreme susceptibility to radiation-induced MBs that is characteristic of CD1*^Ptch1^*^+/−^ mice.

### 3.3. p53 Activity and Cell Cycle Assessment in GCPs in Response to DNA Damage

**Figure 3:**
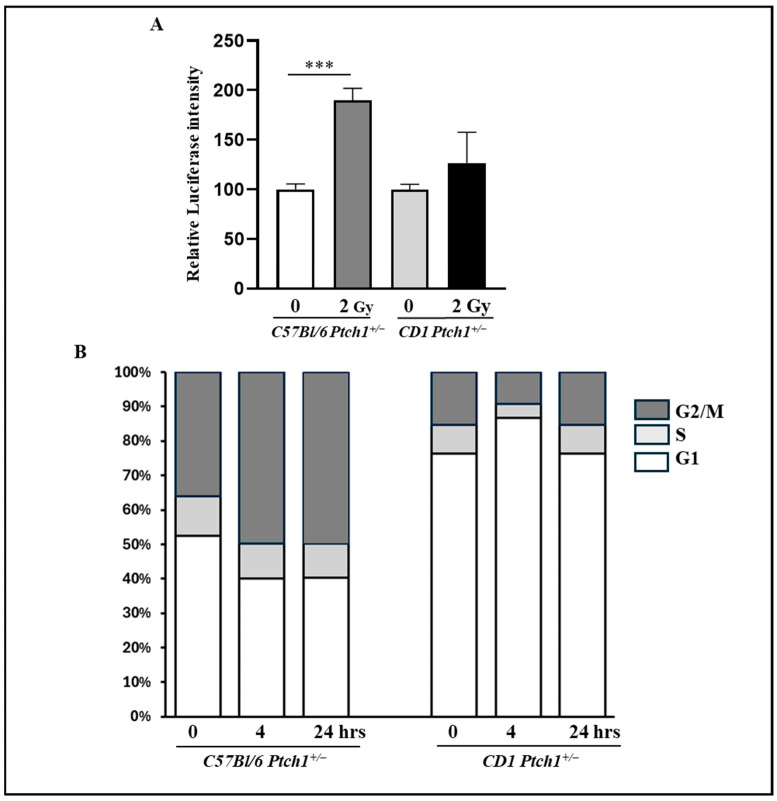
p53 functional assay and flow cytometry cell cycle analysis in GCPs from 2 Gy-irradiated CD1*^Ptch1^*^+/−^ and C57Bl/6*^Ptch1^*^+/−^ mice and relative unexposed controls. (**A**) p53 functional assay on GCPs 2 h post irradiation. The results are expressed as an average of 3 biological replicates ± SD; untreated GCPs^C57Bl-^*^Ptch1^*^+/−^ and GCPs^CD1-*Ptch1*+/−^ were taken as 100. (**B**) Flow cytometry analysis of the cell cycle at different post-irradiation times. The graphs present the mean of three experiments. *** *p* < 0.001.

The p53 tumor suppressor controls cellular homeostasis and genome stability and it is activated in response to irradiation. To assess p53 activity, we performed functional assays with a reporter vector containing p53-responsive elements. Following transfection and irradiation, GCPs^C57Bl-^*^Ptch1^*^+/−^ exhibited a marked significant activation of the p53 protein compared to their unirradiated counterparts at 2 h post-irradiation (189.17 vs. 100; *p* = 0.003). In contrast, similar treated GCPs^CD1-*Ptch1*+/−^ displayed a minor and insignificant modulation (126.71 vs. 100; *p* = 0.2139) under the same conditions (Figure 3A).

We conducted a flow cytometry analysis of the cell cycle. We collected 2 Gy-irradiated GCPs at 4 and 24 h post-irradiation and compared them with their non-irradiated counterparts. GCPs^C57Bl-^*^Ptch1^*^+/−^ exhibited a G2 phase block, shown by an increase in the G2/M population from 36% to 50% at 4 h post-irradiation, which also persisted at 24 h post-irradiation (Figure 3B). Conversely, GCPs^CD1-*Ptch1*+/−^ displayed a G1 phase block at 4 h post-irradiation, demonstrated by an increase in the G1 population from 76% to 82%, which was resolved by 24 h after irradiation (Figure 3B).

Altogether, following 2 Gy irradiation, GCPs^C57Bl-^*^Ptch1^*^+/−^ showed increased p53 activation and a cell cycle block in G2/M compared to GCPs^CD1-*Ptch1*+/−^, which instead exhibited a G1 block, indicative of a genetic background-related difference in the mechanism of radiation-induced cell cycle delay.

### 3.4. Gene Expression Analysis in Spontaneous and Radiation-Induced MBs

To determine the molecular basis of the genetic background-related susceptibility to radiation-induced MBs, we have performed a gene expression analysis of MB tumors (n = 14 for each group), developed in unexposed or irradiated C57Bl/6*^Ptch1^*^+/−^ and CD1*^Ptch1^*^+/−^ mice. 

**Figure 4:**
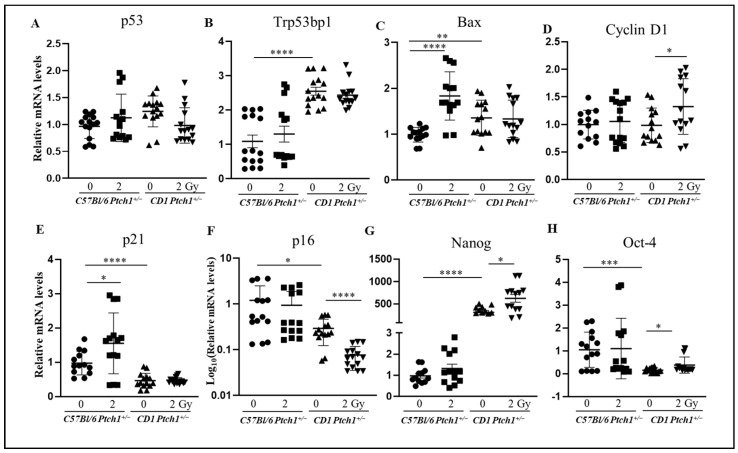
Gene expression analysis in spontaneous and radiation-induced MBs. Evaluation of mRNA expression levels of (**A**) *p53*, (**B**) Trp53bp1, (**C**) *Bax*, (**D**) *Cyclin D1*, (**E**) *p21*, (**F**) *p16*, (**G**) *Nanog*, and (**H**) *Oct-4*. The results are expressed as an average of 14 MBs ± SEM; untreated GCPs^C57Bl-^*^Ptch1^*^+/−^ were taken as 1. The differences were analyzed with a Student’s *t*-test: **** *p* < 0.0001, *** *p* < 0.001, ** *p* < 0.01, and * *p* < 0.5.

To maintain genomic stability, cells have developed a network of signaling pathways to sense and repair DNA damage, including DNA repair mechanisms and cell cycle checkpoints, as well as specialized programs, such as apoptosis and senescence. We examined key genes in radiation response such as p53 and *Trp53bp1*, along with genes involved in apoptosis (*Bax*), control of the cell cycle (*p21* and *Cyclin D1*), senescence (p16), and stemness (Nanog and Oct-4). The expression levels of p53 were not different in irradiated and unirradiated MBs from both strains (Figure 4A).

Like GCPs, a 2.6-fold higher expression of *Trp53bp1* was observed in spontaneous MBs from C57Bl/6*^Ptch1^*^+/−^ compared with MBs from CD*^Ptch1^*^+/−^ mice (*p* < 0.0001) (Figure 4B). Instead, Trp53BP1 expression was comparable in MBs from irradiated and unirradiated C57Bl/6*^Ptch1^*^+/−^ and CD*^Ptch1^*^+/−^ mice.

We next evaluated *Bax* expression and, similarly to GCPs, spontaneous MBs from CD1*^Ptch1^*^+/−^ mice showed a significant 35% increase in *Bax* expression compared to spontaneous MBs from C57Bl/6*^Ptch1^*^+/−^ mice (*p* = 0.0027; Figure 4C). Radiation-induced MBs from C57Bl/6*^Ptch1^*^+/−^ mice showed a significant 80% increase in *Bax* expression compared with spontaneous MBs (*p* < 0.0001), but no discernible changes were observed between spontaneous and irradiated MBs from CD1*^Ptch1^*^+/−^ mice.

We next analyzed cyclin D1 expression to evaluate cell proliferation (Figure 4D). *Cyclin D1* basal expression was comparable in spontaneous MBs from C57Bl/6*^Ptch1^*^+/−^ and CD1*^Ptch1^*^+/−^ mice. While no difference was observed between spontaneous and irradiated MBs in C57Bl/6*^Ptch1^*^+/−^ mice, irradiated MBs from CD1*^Ptch1^*^+/−^ mice showed a significant 20% increase compared with spontaneous MBs (*p* = 0.042).

*p21* mRNA expression is 50% higher in spontaneous MBs from C57Bl/6*^Ptch1^*^+/−^ mice compared to their CD1 counterparts (*p* < 0.0001). Moreover, irradiation significantly increased *p21* expression by 40% in MBs from irradiated C57Bl/6*^Ptch1^*^+/−^ mice compared to spontaneous MBs (*p* = 0.032) (Figure 4E), while it remained unchanged in radiogenic MBs from CD1*^Ptch1^*^+/−^ mice.

*p21* is also involved in the control of senescence along with *p16*^INK4a^. Under baseline conditions, spontaneous MBs from C57Bl/6*^Ptch1^*^+/−^ mice showed 70% higher *p16*^INK4a^ expression levels than their CD1 counterparts (*p* < 0.0156). While no difference was shown in *p16* expression between spontaneous and radiation-induced MBs from C57Bl/6*^Ptch1^*^+/−^ mice, a significant 60% reduction was observed in radiation-induced vs. spontaneous MBs from CD1*^Ptch1^*^+/−^ mice (*p* < 0.0001) (Figure 4F).

We also compared the expression of the stemness genes *Nanog* and *Oct-4* in spontaneous and radiation-induced MBs from the two genetic backgrounds. Similar to GCPs, spontaneous MBs from CD1*^Ptch1^*^+/−^ mice show a 300-fold higher basal expression level of *Nanog* compared to spontaneous MBs from C57Bl/6*^Ptch1^*^+/−^ mice (*p* < 0.0001). A 2-fold increase in Nanog expression was also observed in radiogenic vs. spontaneous MBs from CD1*^Ptch1^*^+/−^ mice (*p* = 0.0284), while no significant changes in *Nanog* were detected between spontaneous and irradiated MBs from C57Bl/6*^Ptch1^*^+/−^ mice (Figure 4G). Similar to GCPs, spontaneous MBs from C57Bl/6*^Ptch1^*^+/−^ mice also showed 6- to 7-fold higher Oct-4 expression than their CD1 counterparts (*p* = 0.0002) (Figure 4H). No significant changes in *Oct-4* expression were detected between spontaneous MBs and those developed in irradiated C57Bl/6*^Ptch1^*^+/−^ mice, while radiogenic MBs from CD1*^Ptch1^*^+/−^ mice showed 2-times higher *Oct-4* expression compared to spontaneous MBs (*p* = 0.0327).

Overall, we demonstrated differential genetic background influences in the expression of six out of eight genes associated with DNA damage response (*Trp53bp1* and *p21*), apoptosis (*Bax*), senescence (*p16*), and stemness (*Nanog* and *Oct-4*), which allow us to discriminate spontaneous MBs developed in C57Bl/6*^Ptch1^*^+/−^ mice from those in CD1*^Ptch1^*^+/−^ mice. We also identified specific genetic background-related features in MBs from C57Bl/6*^Ptch1^*^+/−^ and CD1*^Ptch1^*^+/−^ mice after irradiation. MBs from C57Bl/6*^Ptch1^*^+/−^ mice showed elevated levels of *Bax* and *p21* compared to their spontaneous counterparts. Conversely, radiation-induced MBs in CD1*^Ptch1^*^+/−^ mice displayed reduced senescence (*p16*) and increased proliferation (*Cyclin D1*) and stemness (*Nanog*, *Oct-4*) compared to their spontaneous counterparts, reminiscent of the GCPs’ response to irradiation.

### 3.5. Correlation Between the Expression Levels of Genetic Background-Related and Radiation-Induced Genes and Survival in MB Patients

To expand the translational meaning of our results, we used the R2 Genomic Analysis and Visualization Platform to interrogate a dataset of 331 primary MBs [15] and perform Survival Analyses on the basis of the expression of the significantly modulated genes in spontaneous and radiation-induced murine MBs. We focused on MB patients for a comprehensive survival analysis. Radiotherapy is an elective treatment in the management of MB [16]. We referred to the R2 site (https://hgserver1.amc.nl/cgi-bin/r2/) to access data (16 July 2024) on MB patients.

**Figure 5:**
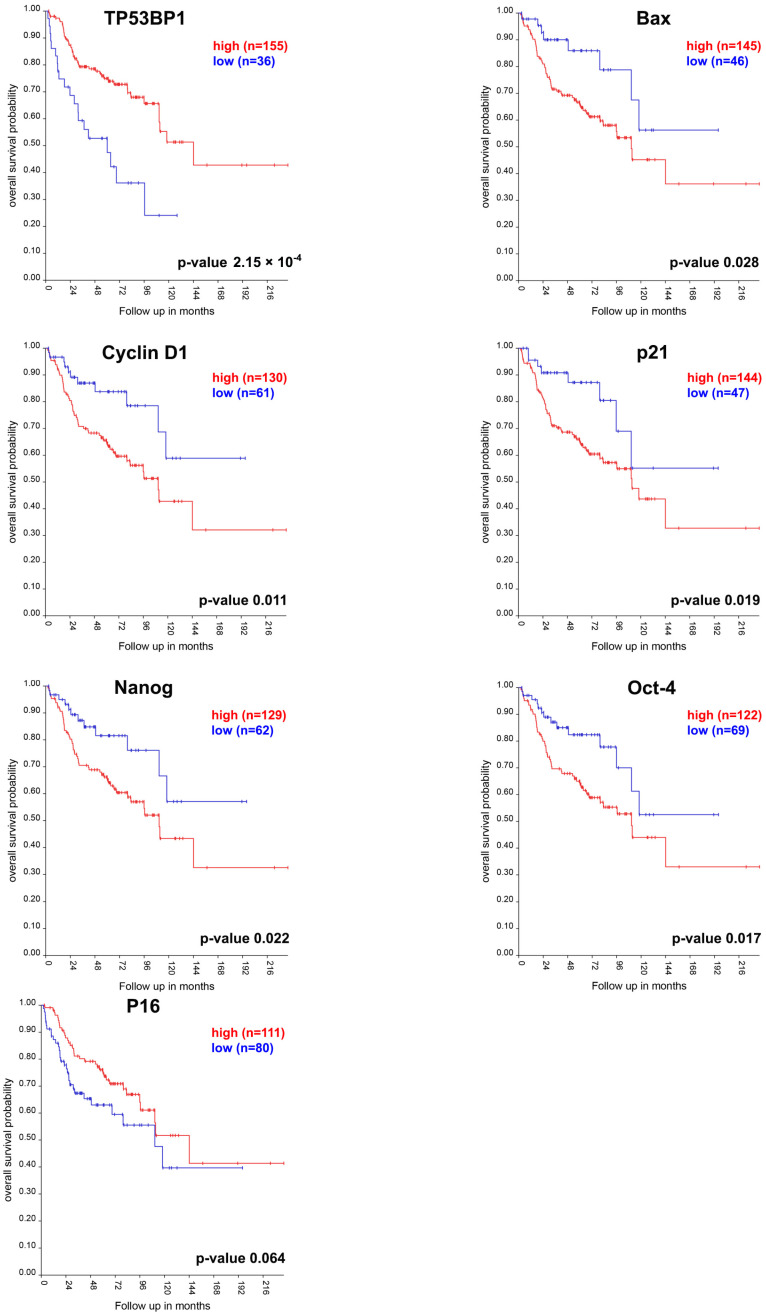
Kaplan–Meier plots showing the impact of gene expression (high versus low levels) on survival in MB patients. Single gene analysis for *TP53BP1*, *BAX*, *CYCLIN D1*, *P21*, *NANOG*, *OCT-4*, and *P16*. Log-rank test *p*-values are indicated at the bottom right of each plot.

Kaplan–Meier survival analysis for MB patients revealed significant survival differences for six of the seven mouse genetic background-dependent genes tested based on their expression levels. Decreased survival was associated with increased expression of *CYCLIN D1* (*p* < 0.011), *P21* (*p* < 0.019), *BAX* (*p* < 0.028), *NANOG* (*p* < 0.022), and *OCT-4* (*p* < 0.017), and with decreased expression of *TP53BP1* (*p* < 0.0002). However, the expression of *P16* did not show a statistically significant correlation with survival (*p* < 0.064) (Figure 5).

The significant difference in the Kaplan–Meier survival analysis between MB patients with high and low expression of genes, which are differentially expressed in mice with varying susceptibility to radiogenic MB induction, highlights the translational relevance of our murine model findings.

### 3.6. MBs’ Response to Irradiation Is Modulated by Genetic Background

**Figure 6:**
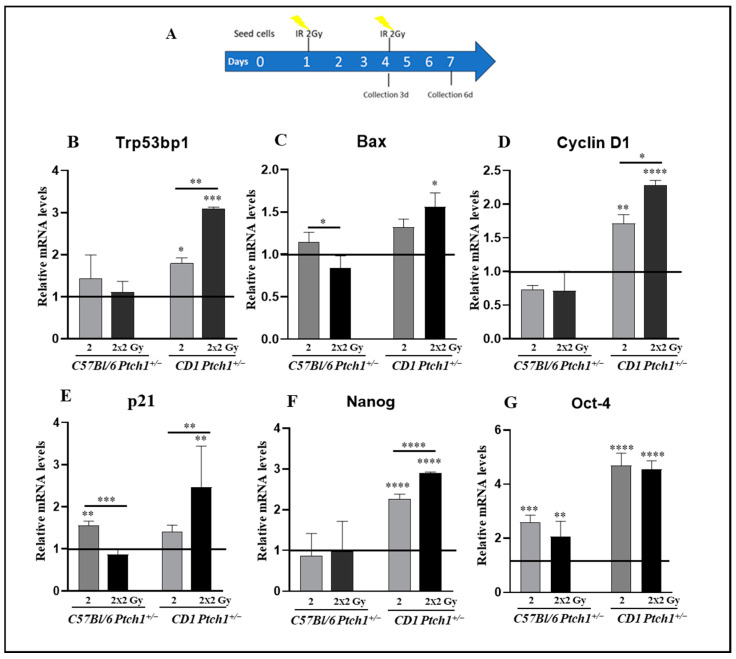
Gene expression in spontaneous MBs from C57Bl/6*^Ptch1^*^+/−^ and CD1*^Ptch1^*^+/−^ mice after one or two 2 Gy X-ray fractions (yellow symbols). (**A**) Experimental scheme. (**B**) Expression levels of *Trp53bp1*, (**C**) *Bax*, (**D**) *Cyclin D1*, (**E**) *p21*, (**F**) *Nanog*, and (**G**) *Oct-4*. The results are expressed as an average of 3 biological replicates and expressed as means ± SE. Unirradiated C57Bl/6^*Ptch1*+/−^ and CD^*Ptch1*+/−^ spontaneous tumours are taken as 1. Differences were analyzed using an ANOVA test. Asterisks above the bars indicate comparisons between the specified groups, while asterisks over the histograms refer to comparisons with the corresponding unexposed control group: show * *p* < 0.05, ** *p* < 0.01, *** *p* < 0.001, **** *p* < 0.0001.

Interestingly, data on murine GCPs and MBs and human MB patients suggested a potential link between gene expression signatures and MB susceptibility. We aimed to determine whether the expression levels of the genes correlated to survival in MB patients (Trp53bp1, Bax, p21, Cyclin D1, Nanog, and Oct4) also influence the radiation response and can predict tumor reactions to irradiation. To investigate further, we studied the response of spontaneous MBs (n = 4 for each group) from C57Bl/6*^Ptch1^*^+/−^ and CD1*^Ptch1^*^+/−^ mice to either a single or two repeated X-ray treatments of 2 Gy, mirroring radiotherapy fractions. MBs were explanted, cultured, and irradiated following the experimental scheme shown in Figure 6A, and then assessed 3 days post-irradiation for the expression of genes associated with DNA damage response (*Trp53bp1* and *Bax*), cell cycle regulation (*p21* and *Cyclin D1*), and stemness (*Nanog* and *Oct4*).

Irradiated MBs from C57Bl/6*^Ptch1^*^+/−^ mice did not show modulation in *Trp53bp1* levels following either single or repeated irradiation compared to unexposed MBs, while MBs from CD1*^Ptch1^*^+/−^ mice displayed a progressive increase (*p* = 0.0065) of 1.8-fold after one fraction (*p* = 0.045) and 3-fold after two fractions (*p* = 0.0001) (Figure 6B).

*Bax* expression was substantially unchanged in MBs from C57Bl/6*^Ptch1^*^+/−^ mice after either a single or two repeated 2 Gy fractions compared to unexposed MBs (Figure 6C), although a significant decrease was observed after two 2 Gy fractions compared to one (*p* = 0.0001). Instead, while a single fraction does not modify the expression levels, irradiation with two fractions induced a significant 1.6-fold increase (*p* = 0.0104) in MBs from CD1*^Ptch1^*^+/−^ mice.

*Cyclin D1* expression was substantially unchanged in MBs from C57Bl/6*^Ptch1^*^+/−^ mice after either a single or two repeated 2 Gy fractions (Figure 6D). Instead, MBs from CD1*^Ptch1^*^+/−^ mice irradiated with one or two fractions of 2 Gy showed progressive and significant (*p* = 0.033) increases of 1.7-fold (*p* = 0.0043) and 2.5-fold (*p* < 0.0001), respectively (Figure 6D), showing a consistent and proliferative gain that underlies a poor prognosis.

Regarding *p21* expression in MBs from C57Bl/6*^Ptch1^*^+/−^ mice, although a significant upregulation was observed after a single 2 Gy fraction, either compared to the unexposed control or to a single 2 Gy fraction (*p* = 0.0050 and *p* = 0.0003), there was not a significant change after two 2 Gy fractions when compared to unexposed MBs. Conversely, a single dose of irradiation produced no significant changes in MBs from CD1*^Ptch1^*^+/−^ mice, while two repeated 2 Gy fractions resulted in a significant increase vs. either untreated mice (*p* < 0.0001) or mice irradiated with one fraction (*p* = 0.0091) (Figure 6E).

Nanog expression levels remained largely unchanged in MBs from C57Bl/6*^Ptch1^*^+/−^ mice when compared to unexposed MBs. Conversely, in MBs from CD1*^Ptch1^*^+/−^ mice, Nanog expression exhibited a consistent and progressive rise (*p* < 0.0001), peaking at a 2.2-fold increase after one fraction (*p* < 0.0001) and reaching a 3-fold increase after two fractions (*p* < 0.0001) (Figure 6F).

In irradiated MBs from C57Bl/6*^Ptch1^*^+/−^ mice, *Oct-4* expression levels increased around two-fold (2.57-fold at 2 Gy, *p* = 0.0003; 2.05-fold at 2 × 2 Gy, *p* = 0.0016) compared to unexposed MBs, while higher increases in *Oct-4* expression level were observed in irradiated MBs from CD1*^Ptch1^*^+/−^ mice (4.68-fold at 2 Gy, *p* < 0.0001; 4.54-fold at 2 × 2 Gy, *p* < 0.0001) compared to unexposed MBs (Figure 6G).

Overall, these results indicate that irradiation leads to a rise in the expression levels of *Trp53bp1*, *cyclin D1*, *Nanog*, and *Oct-4* in ex vivo spontaneous MBs from CD1*^Ptch1^*^+/−^ mice, following either a single dose or repeated exposure with 2 Gy or repeated 2 Gy exposure for *Bax* and *p21*. Notably, this effect in MBs from C57Bl/6*^Ptch1^*^+/−^ mice was only observed for *p21* and *Oct-4*. This indicates that the effect of radiation on tumors is influenced by genetic background factors, suggesting that a patient’s response to radiotherapy might be highly dependent on genetic determinants of individual radiosensitivity.

## 4. Discussion

It is well-known that the genetic background on which a highly penetrant cancer gene is studied can dramatically alter the individual risk of developing a specific tumor [17]. The genetic component may also limit or enhance the effect of exposure to environmental cancer-causing agents [18].

In the present study, we have employed the *Ptch1* heterozygous mouse model bred on two genetic backgrounds with opposite susceptibilities to radiogenic MBs, permissive (CD1) or suppressive (C57BL/6), to assess the early radiation response of GCPs, as well as the tumorigenic consequences of ionizing radiation exposure, and elucidate the biological factors responsible for the differential oncogenic response following irradiation.

This study employs a multifaceted approach that includes: (i) examining the endpoints and mechanisms of cellular radiosensitivity in GCPs, MB cells of origin; (ii) identifying differentially expressed genes in both spontaneous and radiation-induced MBs; (iii) employing bioinformatics analyses to link the expression levels of selected radiation-induced genes with survival outcomes in MB cancer patients; and (iv) investigating the expression of these genes in ex vivo MBs subjected to single or repeated irradiation treatments. These results offer a robust dataset to explore correlations between potential determinants of radiosensitivity. In this manner, we generated an intricate network view, which enabled the identification of genes and pathways associated with susceptibility to radiation-induced tumors.

Of note, Shh signaling activation has been reported to induce replication stress in GCPs, detected through increased γ-H2AX and *Trp53bp1* markers [19]. Such Shh-dependent enhanced replication stress, resulting from increased origin firing and fork velocity and mediated by helicase loading and activation, has been implicated in the loss of the wild-type *Ptch1* allele in tumor-prone GCPs [20]. In fact, reduction of replication origins, through Cdc7 inhibition, in Shh-exposed GCPs blocks replication stress along with MB initiation in tumor-prone mice [20].

Our study reveals that GCPs^C57Bl-^*^Ptch1^*^+/−^ and GCPs^CD1-*Ptch1*+/−^ exhibit distinct responses to DNA damage and apoptosis, impacting tumorigenesis. GCPs^CD1-*Ptch1*+/−^ show higher DNA damage markers (Trp53bp1 and γ-H2AX) post-irradiation than GCPs^C57Bl-^*^Ptch1^*^+/−^, with unexposed GCPs^CD1*Ptch1*+/−^ displaying high spontaneous apoptosis (*Bax* and Annexin V), which only moderately increases after irradiation. This spontaneous apoptosis may clear potentially tumor-prone GCPs with *Ptch1* LOH derived from replicative stress [20], contributing to the lower spontaneous tumor incidence (7.7%) in CD1*^Ptch1^*^+/−^ mice. Conversely, the increase in apoptotic rates in GCPs^C57Bl-^*^Ptch1^*^+/−^ after irradiation may prevent the accumulation of radiogenic DNA damage and reduce oncogenic potential, despite a higher spontaneous tumor incidence (40%). Our data—underscoring the pivotal role of genetic backgrounds in shaping DNA damage responses and tumor suppression mechanisms, with strain-dependent distinct strategies to maintain cellular homeostasis and prevent tumorigenesis—also highlight the importance of the genetic context in therapeutic strategies and risk assessment for radiation exposure and cancer.

The present study shows that growth kinetics, as well as the stemness response of GCPs after irradiation, are strongly influenced by the genetic background, possibly affecting radiosensitivity to MB induction. Both GCPs^CD1-*Ptch1*+/−^ and GCPs^C57Bl-^*^Ptch1^*^+/−^ showed a significant initial decrease in growth after irradiation. However, GCPs^CD1-*Ptch1*+/−^ recovered and surpassed the proliferation rate of unirradiated cells after eight days, while GCPs^C57Bl-^*^Ptch1^*^+/−^ showed persistent slow growth. This difference is likely linked to the expression of stemness genes *Oct-4* and *Nanog*, which were upregulated in GCPs^CD1-*Ptch1*+/−^ but downregulated in GCPs^C57Bl-^*^Ptch1^*^+/−^ after irradiation. This is in line with our previous results demonstrating that irradiation of GCPs^CD1-*Ptch1*+/−^ induces the expansion of the stem-like cell compartment through a Nanog-dependent mechanism [14], linking this to the induction of MBs by irradiation.

In addition, our neurosphere assay results provide further insights into the stem cell dynamics post-irradiation. GCPs^CD1-*Ptch1*+/−^ can grow as neurospheres, and this growth potential is significantly diminished when *Oct4* or *Nanog* is silenced. This contrasts sharply with GCPs^C57Bl-^*^Ptch1^*^+/−^, which are unable to grow in a selective stem cell medium. This supports the idea that stemness genes such as *Oct-4* and *Nanog* are crucial for modulating cell growth following irradiation.

Furthermore, *Oct-4* is known to regulate growth, proliferation, the cell cycle, EMT, and DNA repair [21,22], which aligns with the enhanced recovery seen in GCPs^CD1-*Ptch1*+/−^. Moreover, *Oct4* involvement in radioresistance is supported by clinical findings where *Oct4*-positive head and neck squamous cell carcinoma patients had reduced survival post-radiotherapy compared to Oct4-negative cases [23]. Thus, the impact of the genetic background on GCP recovery and differential expression of stemness genes suggests a mechanism for differing radiosensitivity and potential treatment resistance.

These findings collectively suggest that the amplification of stem-like cells driven by *Oct4* and *Nanog* after irradiation enhances the likelihood of radiation-induced MBs in CD1*^Ptch1^*^+/−^ mice. Conversely, the inability of GCPs^C57Bl-^*^Ptch1^*^+/−^ to form neurospheres, due to the downregulation of these stemness genes, may correlate with resistance to in vivo radiation-induced MBs. This highlights the pivotal role of *Oct4* and *Nanog* in influencing the radiosensitivity and potential for tumor progression in different genetic backgrounds.

Upon radiation exposure, the DNA damage response enhances p53 protein levels in cells primarily by promoting protein translation [24] and inhibiting its degradation [25]. Consistent with this, our functional p53 assay showed a significant increase in active p53 in GCPs^C57Bl-^*^Ptch1^*^+/−^ following ionizing radiation exposure, but not in GCPs^CD1-*Ptch1*+/−^. Notably, there is a known link between p53 and *Nanog* expression, as p53 binds to the *Nanog* promoter and suppresses its expression after DNA damage [26]. This suggests that the increased p53 and low Nanog expression levels observed in GCPs^C57Bl-^*^Ptch1^*^+/−^ may be connected.

In irradiated GCPs^C57Bl-^*^Ptch1^*^+/−^, increased p53 activation was accompanied by a persistent G2/M cell cycle block lasting 24 h. In contrast, GCPs^CD1-*Ptch1*+/−^ exhibited only a weak G1 block, which resolved 24 h post-irradiation. This indicates a genetic background-related difference in the mechanism of radiation-induced cell cycle delay.

In CD1*^Ptch1^*^+/−^ mice, MBs are characterized by the loss of the normal remaining *Ptch1* allele through chromosome deletions, suggesting that genome rearrangements may be key events in MB development [27]. Two distinct DSB repair pathways, homologous recombination (HR) and non-homologous end joining (NHEJ), have been developed in mammalian cells to counteract the harmful genotoxic effects [28]. The cell cycle stage at the time of DNA damage induction influences the choice of DSB repair pathway [29]. Most HR-based DNA repair happens in the S and G2 phases of the cell cycle when an undamaged sister chromatid is available for use as a repair template. NHEJ, taking place when the cells are blocked in G1, does not require homology for DSB repair and corrects the break in an error-prone manner.

On this issue, our previous data, obtained from CD1*^Ptch1^*^+/−^ mice, indicated a prominent role for HR in genome stability, as *Rad54* deficiency increased both spontaneous and radiation-induced MB development in *Ptch1*^+/−^/*Rad54*^−/−^ mice. Instead, loss of NHEJ function led to the suppression of MB tumorigenesis in *Ptch1*^+/−^/*DNA-PKcs*^−/−^ mice through increased DSBs and apoptosis in GCPs, leading to the killing of tumor-initiating cells [30]. It is therefore plausible to speculate that GCPs from CD1*^Ptch1^*^+/−^ mice, which arrest in G1 following irradiation, primarily employ NHEJ for DSB repair. This can lead to extensive genome rearrangements, driving MB oncogenesis. Supporting this, the Nanog protein, which is upregulated upon irradiation in CD1*^Ptch1^*^+/−^, has been identified as a Rad51 inhibitor, blocking HR and promoting NHEJ repair through its binding [31]. Additionally, *Trp53bp1*, upregulated following irradiation in CD1*^Ptch^*^+/−^ mice, binds ubiquitinated histones within chromatin associated with DSBs to promote NHEJ [32,33,34]. Conversely, GCPs^C57Bl-^*^Ptch1^*^+/−^, which markedly and persistently arrest in G2/S after damage, undergo HR repair, thereby preventing the induction of radiogenic MBs.

To determine the molecular basis of differences between C57Bl/6*^Ptch1^*^+/−^ and CD1*^Ptch1^*^+/−^ mice, we also performed gene expression analysis in spontaneous and radiation-induced MBs. Our data highlight the significant influence of the genetic background on the expression of several genes associated with DNA damage response (*Trp53bp1* and *p21*), apoptosis (*Bax*), senescence (*p16*), and stemness (*Nanog* and *Oct-4*) in MBs. These genetic differences help distinguish spontaneous MBs in C57Bl/6*^Ptch1^*^+/−^ mice from those in CD1*^Ptch1^*^+/−^ mice. Furthermore, we observed distinct genetic background-related features in MBs from irradiated C57Bl/6*^Ptch1^*^+/−^ and CD1*^Ptch1^*^+/−^ mice.

In irradiated C57Bl/6*^Ptch1^*^+/−^ mice, MBs exhibited elevated levels of *Bax* and *p21* compared to MBs from unirradiated mice, indicating a heightened apoptotic and DNA damage response. In contrast, radiation-induced MBs in CD1*^Ptch1^*^+/−^ mice showed reduced senescence (*p16*), increased proliferation (*Cyclin D1*), and elevated stemness (*Nanog*, *Oct-4*) relative to spontaneous tumors. These differences reflect the distinct responses of GCPs to irradiation based on genetic background, with CD1*^Ptch1^*^+/−^ mice demonstrating a more pronounced stemness and proliferation response, potentially contributing to increased tumor progression following radiation exposure.

**Figure 7:**
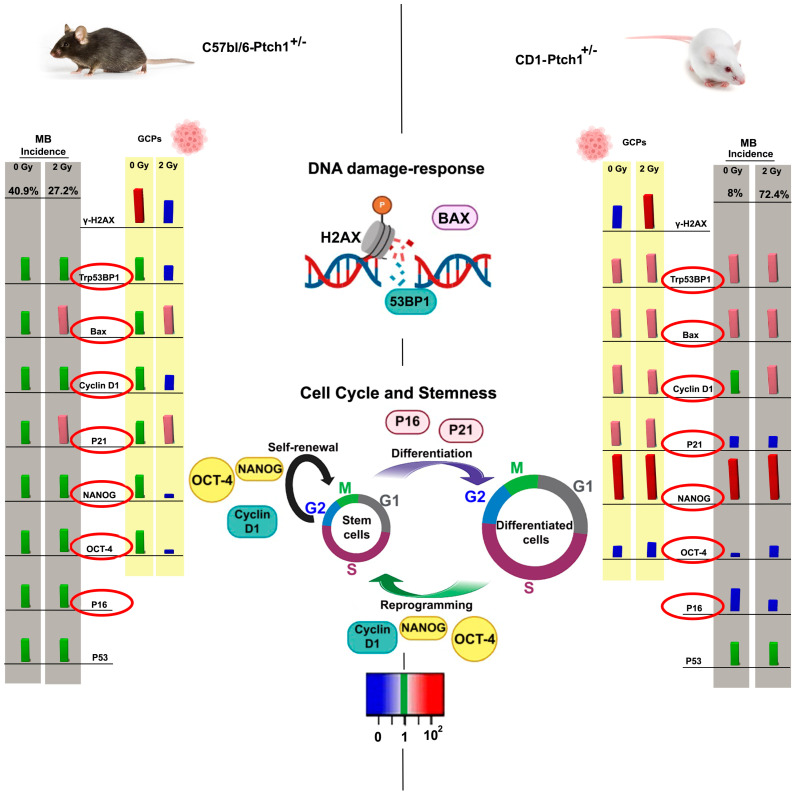
Genetic background-related expression data in GCPs and MBs from *Ptch1*^+/−^ mice. Global recap of the frequencies of spontaneous and radiation−induced MBs (2 Gy) in C57Bl/6*^Ptch1^*^+/−^ (**left**) and CD1*^Ptch1^*^+/−^ mice (**right**) [12], compared to the level of residual DSBs at 24 h (γ-H2AX and Trp53bp1*)* and expression of genes involved in DNA damage response (*Bax*, *p21*, *p16*, and *p53*) and in the cell cycle and stemness (*Cyclin D1*, *Nanog*, *Oct-4*, *p21*, and *p16*). *In vitro* data from sham (0 Gy) and irradiated (2 Gy) GCPs are in the yellow panels, while in vivo data from spontaneous and radiation-induced MBs are in grey. Schematic bars represent the relative expression levels compared to the expression in unexposed C57Bl/6*^Ptch1^*^+/−^ control samples (Spontaneous MBs and GCPs): non-statistically different (green); downregulated genes (*p* < 0.01) (blue); slightly upregulated genes (1.5 ≤ Relative mRNA levels < 4, *p* < 0.01) (pink); strongly upregulated genes (Relative mRNA levels > 10, *p* < 0.0001) (red).

In the scheme of Figure 7, to simplify the comparison of data on GCPs and MBs, we have summarized the frequency of spontaneous and radiogenic MBs in C57Bl/6*^Ptch1^*^+/−^ (left side) and CD1*^Ptch1^*^+/−^ (right side) mice with the expression of gene markers for DSBs (γ-H2AX and *Trp53bp1*), DNA damage response (*Bax*, *p21*, *p16*, and p53), cell cycle, and stemness (*Cyclin D1*, *Nanog*, *Oct-4*, *p21*, and *p16*) in GCPs (yellow panel) and MBs (grey panel). These findings highlight that cellular processes, such as DNA damage response, apoptosis, cell growth, cell cycle regulation, and stemness, critical to determining cellular outcomes after exposure to ionizing radiation are differently regulated in C57Bl/6*^Ptch1^*^+/−^ and CD1*^Ptch1^*^+/−^ mice. Therefore, we proposed a potential panel of genes whose expression varies with genetic background and might influence the susceptibility to radiogenic MBs. These signature genes include *Nanog*, *Trp53bp1*, *Bax*, *Cyclin D1*, *p21*, and *p16* (red circle in Figure 7), which are believed to affect radiation response.

Survival analysis performed using the R2 Genomic Analysis and Visualization Platform, interrogating a dataset of 331 primary MBs with clinical pathological annotation and based on genes differentially expressed in our radiation-induced murine MBs (i.e., *TRP53BP1*, *BAX*, *CYCLIN D1*, *P21*, *NANOG*, *OCT-4*, and *P16*), revealed significant decreases in survival among MB patients with high expression levels of *CYCLIN D1* (*p* < 0.011), *P21* (*p* < 0.019), *BAX* (*p* < 0.028), *NANOG* (*p* < 0.022), and *OCT-4* (*p* < 0.017), and with decreased expression of *TP53BP1* (*p* < 0.0002), strongly supporting the translational relevance of the results obtained from our mouse models.

Our findings, derived from the genetic backgrounds of susceptible or resistant radiation-induced MBs and validated through bioinformatics survival analyses of MB patients, support the presence of a potential gene expression signature. This signature, comprising *TRP53BP1*, *P21*, *BAX*, *CYCLIN D1*, *OCT-4*, and *NANOG*, appears to influence tumor response to radiation. Notably, in ex vivo murine spontaneous MBs, the expression of *Trp53bp1*, *Bax*, *Cyclin D1*, *p21*, and *Nanog* gradually increases following either single or repeated radiation exposure in the CD1 genetic background but not in the C57Bl/6 background. This observation suggests that such a genetic background-related signature could be a valuable tool for predicting tumor response to radiation therapy.

Beyond predictive work, a better understanding of protective DDRs—whose functioning obviously confers radioresistance—is crucial for targeting DNA damage repair in tumors, which has become an attractive strategy in radiotherapy and provides critical therapeutic opportunities for overcoming tumor radioresistance [35]. Identifying treatment-resistant genotypes and therapeutic targets that may influence tumor response is at the center of current radiation biology research.

## 5. Conclusions

This study underscores the critical role of genetic background in modulating radiation sensitivity and biological outcomes, as demonstrated by the differential regulation of cellular processes in C57Bl/6*^Ptch1^*^+/−^ and CD1*^Ptch1^*^+/−^ mice following irradiation. C57Bl/6^Ptch1+/−^ mice exhibit a stringent DNA damage response, characterized by elevated apoptosis rates and cell cycle arrest, potentially offering protection against cancer at the cost of slower tissue recovery. Conversely, CD1*^Ptch1^*^+/−^ mice show a more permissive response, facilitating quicker recovery but potentially increasing genomic instability and cancer risk. These findings provide insights that could help identify genetic susceptibilities relevant to radiation exposure, with potential implications for future assessments in human populations.

## Data Availability

The dataset for the human medulloblastoma analysis is available on the R2 Genomic Analysis and Visualization Platform (Amsterdam UMC, https://hgserver1.amc.nl/cgi-bin/r2/; accessed on 16 July 2024).

## Supplementary Materials

The following supporting information can be downloaded at: https://www.mdpi.com/article/10.3390/cells13221912/s1, Figure S1: Two-group segregation of MB patients based on gene expression levels.
