# Supplementary figures and images for: Dissecting the Impact of Genetic Background on Oncogenic Response to Radiation Exposure in the Ptch1+/− Mouse Model
Source: Cells. 2024 Nov 19;13(22):1912. doi: 10.3390/cells13221912 (PMC11593216; doi:10.3390/cells13221912)

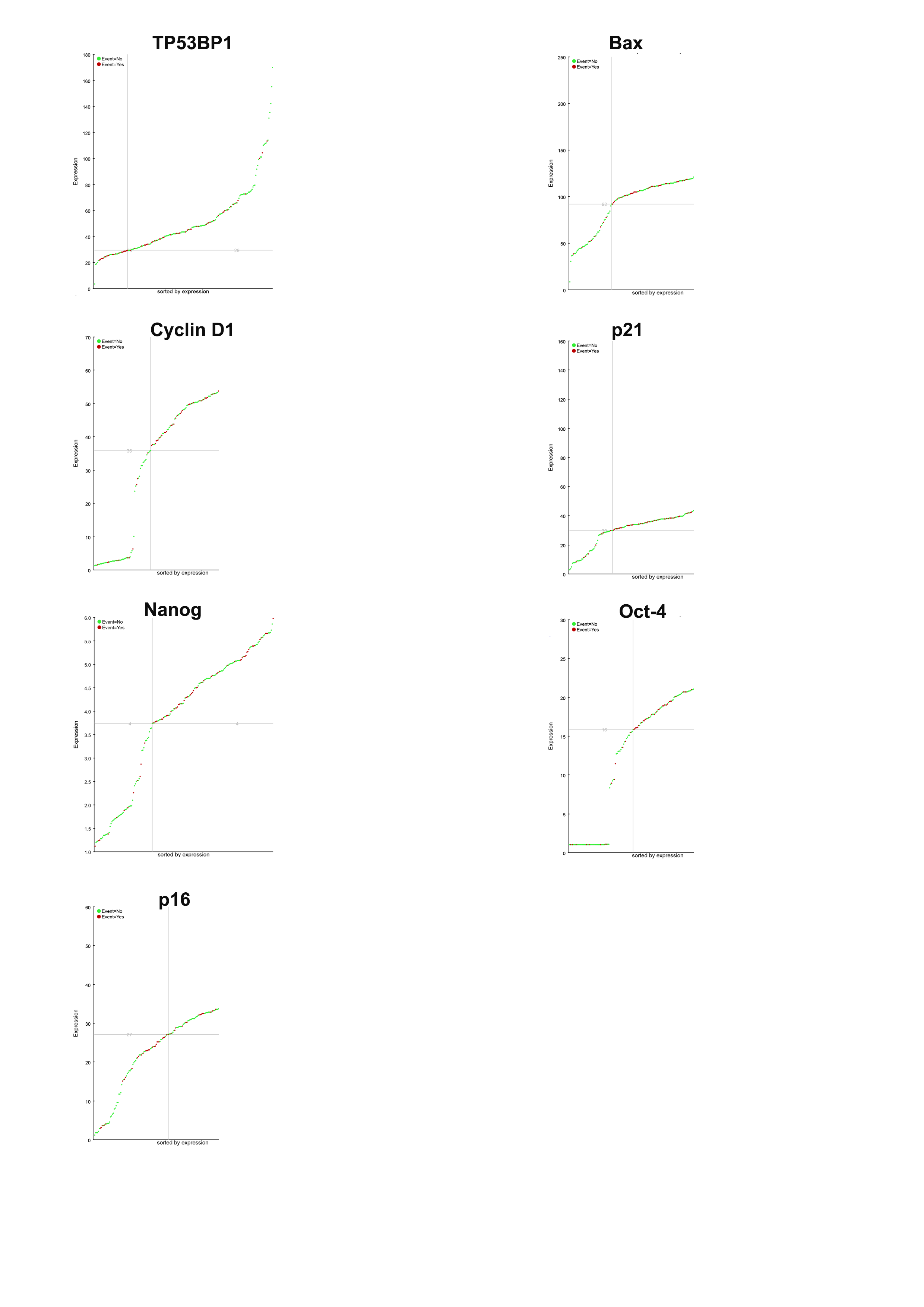

Supplement: Supplementary file 1 [file cells-13-01912-s001.zip › cells-3269236-supplementary.tif]
